# Transcranial Direct Current Stimulation Effects on Athletes’ Cognitive Performance: An Exploratory Proof of Concept Trial

**DOI:** 10.3389/fpsyt.2016.00183

**Published:** 2016-11-30

**Authors:** Davimar M. M. Borducchi, July Silveira Gomes, Henrique Akiba, Quirino Cordeiro, José Henrique M. Borducchi, Lívia Stocco Sanches Valentin, Gabrielle M. Borducchi, Álvaro Machado Dias

**Affiliations:** ^1^Clinical Neuroscience Laboratory, Department of Psychiatry, Federal University of São Paulo, São Paulo, Brazil; ^2^Clinical Neuromodulation Laboratory, Santa Casa of São Paulo Medical Science College, São Paulo, Brazil

**Keywords:** tDCS, cognitive performance, athletes, ethics, doping

## Abstract

Among the 2016 Olympic and Paralympic Games’ unforgettable moments, one could not overlook performances by Phelps and Bolt, which challenge old premises about the maximum extension of individual supremacism in ultracompetitive modalities and the doping scandals. Different media channels resonated these two trends, with an unseen rise on discussions about traits and practices that may set ultrahigh performance athletes apart from the more ordinary ones. Yet, some key issues remain undebated. This paper aims to add to this debate, with a proof of concept trial, which investigates whether transcranial direct current stimulation (tDCS) may serve as an aid for professional athletes. Ten professional athletes of three different modalities (judo, *N* = 4 athletes, swimming, *N* = 3 athletes, and rhythmic gymnastics, *N* = 3 athletes) received anodal stimulation (2 mA) for 20 min on the left dorsolateral prefrontal cortex for ten consecutive weekdays. We observed a positive effect of tDCS in their cognitive performance, including a significant improvement in alternated, sustained, and divided attention and in memory scores. We also observed a decrease in Beck Depression Inventory scores (4.50 points) in this non-clinical population. These preliminary results suggest that tDCS sessions may translate into competitive advantages for professional athletes and recommend the deepening of the discussion on its ethical use in sports, which is ultimately tied to the wider debate around the risks and opportunities that neuromodulation brings to the table.

## Introduction

The 2016 Olympic Games ignited throughout the media the enduring discussion about traits and practices that may set ultrahigh performance athletes apart from the more ordinary ones. On one side, phenomena like Phelps and Bolt proved that what some skeptical sports analysts thought to be impossible (specially about prolonged supremacy in highly competitive modalities) is indeed possible; whereas, on the other side, the world watched stupefied to history’s widest and most publicized doping scandal ever.

In one way or the other – either clean or not – a consensus of the current days is that in the far majority of modalities (with exceptions like weight lifting), super-performers outrival their opponents through the development of competences in three information processing domains (1): perception (2), cognition and visual imagery (3), and action (4), to which affective control is added as a mean to accomplish behavioral maximization, under a premise that elite athletes are mentally and emotionally strong (5). In that same sense, specialized practices are habitually divided in the former three and often accompanied by stress management programs, whereas ethical coaching is much about enhancing them, without the use of illegal practices or substances. However, in the last 2 or 3 decades, the progress in performance of elite athletes has reached more stable levels, when compared to the beginning of the century (6).

Interventions, as meditation and neural brain stimulation, have been investigated toward their effects improving cognition and performance in sports, although the number of peer-reviewed publication still incipient. Meditation techniques, including mindfulness, breathing exercises, and mental imagery, are associated with increasing flow experience (7), to change heart rate variability (8), and reduce oxidative stress (9) in community sport practitioner and professional athletes, respectively.

Transcranial direct current stimulation (tDCS) is a low cost, non-invasive, and painless neural modulation technique that uses low intensity direct electrical current to stimulate gross-defined cortical sites (low cost, no neural navigation used), inducing increased/decreased thresholds for action potentials (10). It has been reported to improve behavioral performance in a diverse array of cognitive domains: attention, object recognition and memory (11), reaction time (12), and motor skill acquisition (13); moreover, it is also believed to support stress management through physiological control of the autonomic system (14), which may translate into performance gains in many sports. Recently, Reardon (15) reported preliminary results from other groups about the effects of tDCS reducing perception of fatigue and increase in strength of professional athletes. In that sense, it brings two types of issues to the table: whether it could serve as an aid for professional athletes and whether it should be considered as doping and, hence, prohibited.

The latter is not a simple question. The World Anti-doping Agency (WADA) states that something should be considered as doping if it satisfies at least two of their three factor list, which includes (1) performance enhancements, (2) violations of the spirit of sport, and (3) personal risks. Following the premise that tDCS is a safe and sound technique that may enhance performance in the above domains, the conundrum may be reduced to establishing whether its application represents a violation of such spirit. As discussed in a recent report, sleeping in a tent in high altitudes to increase red blood cell levels is not considered doping, in contrast to hormone intake for the same basic purpose (16, 17). Adding to that, there is no easy way to know whether someone underwent tDCS stimulation during training periods or even right before a competition.

So that is why the former question of whether tDCS can actually produce information processing and psychological gains in sport is so important. More than merely generalizing previously observed results to a different non-clinical population, it can establish the ground for a huge debate, which may change the way the society interprets doping and, most importantly, how authorities should respond in terms of screenings and investigations. With the aim of subsidizing the field in this regard, we designed an open label, proof of concept tDCS trial, with professional athletes in pre-competition preparation (which is their most critical period of training).

## Materials and Methods

With the approval of the local ethical committee, ten athletes (eight males) with ages between 18 and 29 years old (Mean = 23.6 SD = 3.65), at least high school education level (high school: *n* = 1, undergradute: *n* = 4, and graduate: *n* = 5), of three different modalities (judo, *n* = 4 athletes, swimming, *n* = 3 athletes, and rhythmic gymnastics, *n* = 3 athletes) from national-level professional leagues signed an informed consent and received 20 min of 2 mA anodal stimulation for ten consecutive weekdays. Anode was placed on the left dorsolateral prefrontal cortex (DLPFC, F3) and cathode in the contralateral area (F4), according to the 10–20 international electrode positioning system. Stimulation was delivered by a specialized device (Ibramed) with a 25 cm^2^ square rubber electrodes in a saline-soaked sponge. Our choice for DLPFC relied on previous literature reporting cognitive improvement following its stimulation (18).

Only professional athletes, according to the guidelines of Brazilian law n. 9.615/98 (known as *Lei Pelé) were included. The law states that professional athletes are those that are enrolled in professional leagues and are financially tied to sponsors or teams*. A physiotherapist confirmed that none of the athletes suffered injuries in the month before the intervention, and that none were under any type of medical or psychological treatment. None of the athletes reported previous psychiatric or neurological disorders and was not under any medication and all kept their basic training routines to the end of the trial.

In this study, we used a neuropsychological battery to assess cognitive changes after tDCS stimulation in a cohort of professional athletes. The battery was performed immediately before the season’s start (baseline) and 1 week after the end of the tDCS trial. The cognitive functions assessed were memory, attention, and speed of thought.

Transcranial direct current stimulation impacts on phonological and visual working and long-term memories were accessed with TEM-R (Recognition Memory test). In its first phase, TEM-R presents a list of 34 items (16 images and 18 words) to be memorized and recalled in 2 successive attempts. Next, it presents a visual search task and a long-term evaluation, with a delayed recall (25 min) of a list of words and of figures where the participant should match the items on previous list with a similar list composed of the original 34 target stimuli and 15 errors (19).

Attention was evaluated using the TEACO-FF (Concentrated Attention Test), TEALT (Alternated Attention Test), and TEADI (Divided Attention Test), which assesses sustained, alternated, and divided attention. These are graphical tasks, in which the individual has to identify images that are equal to a model among many distractors within a fixed timeframe; performance is based on correct identification, omissions, and incorrect identifications. TEACO-FF has 500 stimuli (320 distractors) and a time limit of 240 s. TEALT uses the same principle, nevertheless, instead of using the same model throughout the whole task, each line has a different model; it is comprised of 352 stimuli (224 distractors) and a time limit of 180 s. TEADI employs the same principle, nevertheless, 3 models are used instead of 1 as in TEACO-FF; it is comprised of 450 stimuli (270 distractors) and a time limit of 300 s (20, 21).

Depression and anxiety symptoms were also screened, using Beck Anxiety Inventory (BAI) and Beck Depression Inventory (BDI). The former consists of 21 questions that track anxiety symptoms using a four-point scale. The Brazilian validation norm sates that moderate anxiety symptoms are presented in individuals who score higher than 20 points (20, 21). BDI consists of 21 questions that track depressive symptoms, using a 0–3 scale. The Brazilian validation norm states that depression is “probably present” in individuals scoring 18 points and up (22, 23); we decided to investigate the effects of the technique on BDI, as professional athletes have an increased risk of becoming depressed after contusions and in light of performance failures (5).

Baseline and post stimulation performance were compared using Wilcoxon Signed Rank Test, suitable for small-related sample comparison. Statistical analyses were performed using SPSS 21. All tests were two tailed and with the significance level of α = 0.05.

## Results

Positive effects of tDCS were observed on mood and cognitive performance dimensions of the athletes (see Table 1). BDI scores decreased by 4.50 points (128.57%) due to the intervention. Alternated, divided, and sustained attention, respectively, showed an increase of 29.5, 5, and 14.5 percentile ranks, whereas recognition memory revealed an improvement of 20 percentile ranks. No performance decline was found in any domain, and no side effects were reported by the athletes or staff team. The non-parametric correlation between the changes on the scores for measure (post intervention scores minus pre intervention scores) was calculated, nevertheless no significant correlation was found. Results are presented in Table 2.

**Table 1:** **Wilcoxon-test comparing performance pre and post stimulation**.

	Pre-stimulation median	IQR^a^	Post-stimulation median	IQR^a^	Test statistics	*p*-value^b^
BAI score	0.00	0	0.00	0	−0.447^c^	1.000
BDI score	8.00	8	3.50	5	−2.810^c^	0.002
Alternated attention^e^	56.00	25	85.50	11	−2.497^d^	0.010
Divided attention^f^	75.00	9	80.00	9	−2.091^d^	0.037
Sustained attention^g^	69.00	6	83.50	8	−2.668^d^	0.004
Recognition memory^h^	50.00	53	70.00	70	−2.226^d^	0.031

*^a^Interquartile range*.

*^b^Two tailed exact test*.

*^c^Based on negative ranks*.

*^d^Based on positive ranks*.

*^e^TEALT*.

*^f^TEADI*.

*^g^TEACO-FF*.

*^h^TEM-R*.

**Table 2:** **Spearman correlation of the difference on each measure, pre, and post stimulation**.

	BAI score	BDI score	Recognition memory^a^	Divided attention^b^	Sustained attention^c^
BDI score	0.138				
	*p* = 0.686				
Recognition memory^a^	−0.139	−0.257			
	*p* = 0.683	*p* = 0.445			
Divided attention^b^	−0.101	0.163	−0.146		
	*p* = 0.767	*p* = 0.632	*p* = 0.669		
Sustained attention^c^	0.541	−0.189	0.468	0.265	
	*p* = 0.086	*p* = 0.578	*p* = 0.146	*p* = 0.431	
Alternated attention^d^	0.405	−0.349	−0.075	0.269	0.346
	*p* = 0.217	*p* = 0.293	*p* = 0.826	*p* = 0.424	*p* = 0.297

*^a^TEM-R*.

*^b^TEADI*.

*^c^TEACO-FF*.

*^d^TEALT*.

## Discussion and Conclusion

Elite athletes outperform the ordinary due to their physiological traits and their techniques. However, among super-athletes, the mental attributes seem to be the golden key (1). Concerns about mental health and improvement of cognitive abilities have increased, but there is still notable lack of the knowledge in this field. According to the current literature (24), high performance athletes are more vulnerable than the general population to mental disorders, especially anxiety and depression. Our study showed that tDCS interventions can increase cognitive performance and diminish depression scores in professional athletes, which in our point of view may contribute to performance gains, greater well-being, and faster recovery. In other words, our results are in line with reports that have shown that tDCS is an effective non-pharmacological treatment for major depressive disorders (25, 26) and should be taken as an important finding, even considering that our sample did not reach baseline levels for being diagnosed with depressive disorder, as bad mood may be strongly counterproductive for this cohort.

Whether brain stimulation should be classified as doping should be carefully debated, without excluding the perspective of defining that in a modality and context-specific fashion, as argued by Davis in this very journal (17); for instance, it is probably not ethic for a swimmer to electrically induce analgesia just before a competition, whereas it may be acceptable for post-competition recovery. As a suggestion, this debate should draw on differences between passive (i.e., tDCS) and active (i.e., meditation) techniques to induce changes to brain functioning (8, 9).

To our knowledge, this is the first report of the effects of tDCS protocol on elite athletes (in pre-competition periodization training, which makes it even more especial). Results are inspiring, but the small sample size, associated with the lack of a sham group, call for caution in generalizing these findings, at this moment. It is worth noting that the latter issue separates this trial from the higher standards in experimental designs in the fields of applied neuroscience and psychology; that said, one could argue that the subjects that were enrolled did not know what to expect from the intervention and that enhancements in cognitive processing (extending beyond working memory gains) are in line with what has been shown by a plethora of double-blind controlled trials, targeting clinical populations (12).

The perspective that tDCS may translate into competitive advantages for professional athletes should not be ignored in the field of sports, ethics, and neuromodulation, which is expanding dramatically under the premise that it has serious potential to popularize mental health care and enhance overall human well-being.

## Ethics Statement

The study was approved by the ethical committee from Federal University of Sao Paulo, CAAE: 34015614.0.0000.0082.

## Author Contributions

All authors contributed to the manuscript and agreed with the content of the work: DB contributed organizing the research, selecting the athletes and writing the paper; JG contributed on the literature research, collecting data and writing the paper; HA contributed in the data analysis and writing the paper; QC contributed in the research design and writing the manuscript; JB contributed selecting and stimulating the athletes; LV contributed defining the instruments methodology and collecting the data; GB contributed selecting and stimulating the athletes; ÁD contributed managing the research team, analyzing the data and writing the paper.

## Conflict of Interest Statement

The authors declare that the research was conducted in the absence of any commercial or financial relationships that could be construed as a potential conflict of interest.
